# Anethole, a Medicinal Plant *Compound,* Decreases the Production of Pro-Inflammatory TNF-α and IL-1β in a Rat Model of LPS-Induced Periodontitis

**Published:** 2014

**Authors:** Janet Moradi, Fatemeh Abbasipour, Jalal Zaringhalam, Bita Maleki, Narges Ziaee, Amin Khodadoustan, Mahyar Janahmadi

**Affiliations:** a *Department of Periodontics, Dental School, Hamadan University of Medical Sciences, Hamadan, Iran.*; b *Dental Faculty, Semnan Unversity of Medical Sciences, Senman, Iran. *; c *Neurophysiology Research Centre (NPRC) and Department of Physiology, Medical School, Shahid Beheshti University of Medical Sciences, Evin, Tehran, Iran. *; d *Private Practice in Periodontics and Implant Surgery, Hamadan, Iran. *

**Keywords:** Trans-anethole, LPS-induced periodontitis, Ketoprofen, Anti-inflammatory, IL-1β, TNF-α

## Abstract

Periodontitis (PD) is known to be one of most prevalent worldwide chronic inflammatory diseases. There are several treatments including antibiotics for PD; however, since *drug resistance is* an increasing problem, new drugs particularly derived from plants with fewer side effects are required. The effects of trans-anethole on IL-1 β and TNF-α level in a rat model of PD were investigated and compared to ketoprofen.

Eschericia coli lipopolysaccharide (LPS, 30 µg) was injected bilaterally into the palatal gingiva (3 µL/site) between the upper first and second molars every two days for 10 days in anesthetized rats. Administration of either trans-anethole (10 or 50 mg/Kg, *i.p*.) or ketoprofen (10 mg/Kg, *i.p*.) was started 20 minute before LPS injection and continued for 10 days. Then, IL-1β and TNF-α levels were measured in blood samples by ELISA at day 0 (control) and at day 10.

Anethole at both concentrations significantly suppressed IL-1β and TNF-α production when compared to LPS-treated rats. The suppressive effects of anethole on LPS-induced pro-inflammatory cytokines were almost similar as seen with ketoprofen.

In conclusion, the present results suggest that anethole may have a potent inhibitory effect on PD through suppression of pro-inflammatory molecules; therefore it could be a novel therapeutic strategy for PD.

## Introduction

Periodontitis (PD) is one of most prevalent worldwide chronic inflammatory diseases characterized by the host-mediated destruction of soft and hard periodontal tissue (1). PD, which is known to be a risk factor for several systemic diseases(2, 3, 4) including cardiovascular diseases, primarily initiated by a number of putative pathogenic bacterial infections. It has also been reported that pro-inflammatory cytokines and chemokines play an important role in the pathogenesis of PD (1, 5, 6, 7). Among cytokines, IL-1β and TNF-α are essential in the development and progression of periodontitis, as it has been shown that their antagonists inhibit the inflammatory response in experimental PD (8). 

There is a close relationship between the high incidence of oral diseases and microorganisms and because of growing antibiotic bacterial resistance, toxic and harmful side effects associate with use of some common antibacterial agents; there is a need for alternative treatment options and therapies that are *effective* and safe and affordable such as herbal therapies (9, 10, 11).Therefor, in this study, the anti-inflammatory effect of anethole in an animal model of periodontitis induced by lipopolysaccharide (LPS) was evaluated.

Phytotherapic compounds particularly those that are containing terpene have been suggested to have anti-inflammatory effects because they can inhibit TNF-*α *(12, 13) and IL-1β production (14). Anethole is a monoterpene position isomer and it is the main constituent of essential oils from aromatic plants including anise, star-anise, and fennel (12, 15). Anethole is used in food and pharmaceutical industries and the United States Food and Drug Administration (FDA-US) has issued its safety certification (12). It has also experimentally shown that anethole has no toxicity at low doses (16) and it is considered non-genotoxic and non-carcinogenic and, therefore, quite safe (17, 18).

Trans-anethole exerts anti-metastatic activity (19), anti-oxidative (17), antimicrobial and antiviral (20), anti-inflammatory (12, 21) properties. It has also been shown that trans-anethole can modify Ca^2+^ and Ca^2+^-activated K^+^ channels function (22).

Based on the above findings, in the present study an attempt was made to investigate the inflammatory potential of trans-anethole in a rat model of periodontitis induced by lipopolysaccharide (LPS). LPS is a potent immune stimulator that induces the release of pro-inflammatory cytokines (*e.g*. IL-1β and TNF-α) and thereby causes acute inflammatory responses (23, 24). IL-1β and TNF-α are both important inflammation markers in the blood serum. After LPS injection, the circulating TNF reaches to its peak within 90 min before of IL-1 (25). LPS has also been shown to induce osteonecrosis (26) Anethole has been shown to block both early and late cellular responses to TNF (27).

## Experimental

*Animal*

Male Wistar rats (180-220 g) were used in the present experiments. The animals were housed under controlled temperature of 23 ± 2 ^o^C and a 12 h light/dark cycle, with free access to tap water and rat chow**. **All procedures were approved by the Ethical Committee for Animal experimentations of Shahid Beheshti University of Medical Sciences. Thirty rats were randomly divided into the following experimental groups (5 rats per group): (1) control; (2) LPS; (3) LPS + dimethyl sulfoxide (DMSO, sham operated group); (4) LPS + trans-anethole (10 mg/Kg, Sigma); (5) LPS + trans-anethole (50 mg/Kg); (6) LPS +Ketoprofen. 

*Model of periodontitis*

The model of periodontitis was done as described by Guimarães and colleagues in 2012 (34). Briefly, rats were anesthetized with ketamine hydrochloride (50 mg/Kg, *i.p*.) and xylazine hydrochloride (5 mg/Kg, *i.p*.), then were fixed on his back. Eschericia coli LPS (30 µg, Sigma, UK) diluted in PBS was injected bilaterally into the palatal gingiva (3 µL/site) using a 10 µL Hamilton micro-syringe between the upper first and second molars every two days for 10 days (a total 5 injections and 150 µg of LPS in each site). Intra-peritoneal administration of either trans-anethole, DMSO (as a vehicle) or ketoprofen (10 mg/Kg) started 20 minute before LPS injections. 

*Blood sampling and cytokines measurement*

After 10 days, blood samples were collected from the retro-orbital sinus of rats under ketamine/xylasine anesthesia into heparin-coated micro-capillaries, and then animals were sacrificed at the end of the experiment (28). The samples were centrifuged and stored at -70 ^o^C. All blood samplings were performed simultaneously for each group (8:00 to 8:15 AM). IL-1β and TNF-α in serum samples were measured with a rat standard ELISA kit (Abcam, UK) at day 0 (control) and at day 10. All plates were pre-coated with either IL-1β or TNF-α antibody, standards, controls and experimental samples were added. All procedures including washing, adding of antibodies, substrate and stop solutions and analyses were done according to the manufacturer's instructions. The plates were read with a micro-plate reader set to 450 nm.

*Statistical analysis*

The data were analysed with one way ANOVA followed by the post-hoc Tukey's test**. **Results are expressed as means ± SEM of five rats. A P value of 0.05 was considered as the limit for statistical significance.

## Results

**Figure 1:**
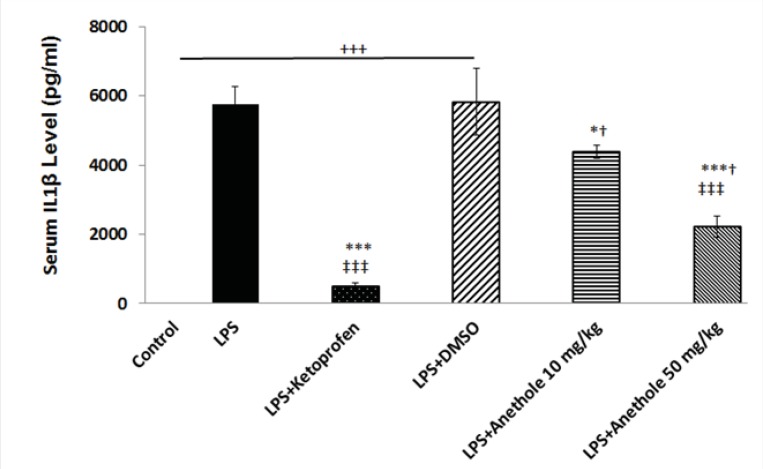
Variation of Serum IL1β level in different study groups

**Figure 2:**
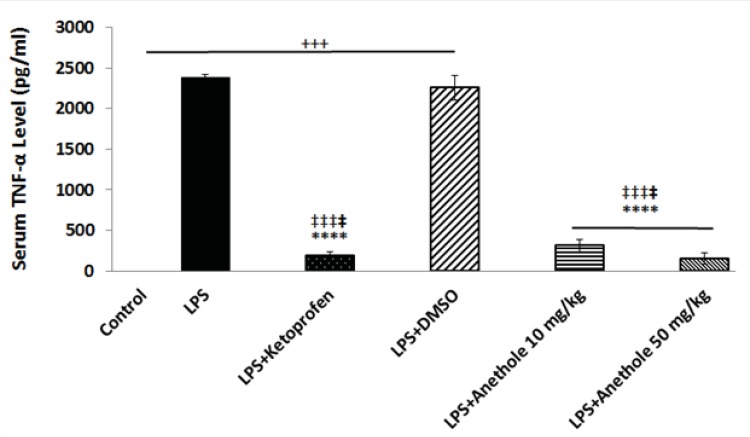
Changes in serum TNF-α level in different study group in a rat model of LPS-induced periodontitis

In the present study, it was determined whether trans-anethole, the chief constituent of several essential oils, suppresses cytokine production in a rat model of periodontitis induced by LPS. Therefore, plasma levels of IL-1β and TNF-α were measured in rats receiving either LPS alone or in combination with intra-peritoneal injection of trans-anethole. Then, the results were compared with those that received LPS plus ketoprofen (*i.p*.), as an anti-inflammatory agent. Administration of LPS into the gingiva between the upper first and second molars significantly increased IL-1β and TNF-α levels (Figures 1 and 2). The plasma level of IL-1β was significantly lower in PD rats receiving concomitant anethole 10 mg/Kg compared to LPS-injected alone group. Although, injection of anethole at 50 mg/Kg led to a further decrease in the IL-1β level, it was still significantly higher than those treated with ketoprofen (Figure 1). Intra-peritoneal injection of 10% DMSO, as the vehicle of anethole, in LPS-treated rats had no effect on theIL1-β level when compared to LPS-treated alone rats (Figure 1).

Injection of LPS into the gingival between the upper first and second molar induced a significant increase in the plasma level of IL-1β. DMSO, as a vehicle, did not affect the increased level of IL-1β due to LPS treatment. Treatment with ketoprofen, as an antiinflamatory drug and anethole both attenuated the inflammatory response induced by LPS as evidenced by a significant reduction in the IL-1β concentration. 

+, *,†, ‡, represents a statistically *significant differences* compared to the control, LPS, LPS+Keto, LPS+DMSO groups, respectively (by *one way ANOVA followed* by Tukey's post hoc).

The plasma level of TNF-α was also measured to assess whether trans-anethole treatment was associated with reduce inflammation in LPS-induced periodontitis. LPS injection alone resulted in a significant increase in the concentration of TNF-α (Figure 2). Anethole treatment, at both doses, caused a significant decrease in the TNF-α level and suppressed the inflammatory response induced by LPS, although this effect was more pronounced at dose 50 mg/Kg. The anti-inflammatory effect of anethole was the same as the effect of ketoprofen (Figure 2). Treatment with DMSO did not attenuate the LPS-induced increase in the plasma level of TNF-α.

A significant increase in serum TNF-α level was observed after LPS administration alone into the gingival of the upper first and second molars, when compare to control rats; whereas anethole treatment, both at lower and higher concentrations, reduced significantly the TNF-α level in the plasma of rats receiving also LPS, which was identical to the effect of ketoprofen. 

## Discussion

Tissue destruction in periodontitis results from the inflammatory response to the microbial products and cytokines including IL-1β and TNF- α. Lipopolysaccharide, a component of the bacterial cell wall is involved in the pathogenesis of several inflammatory diseases as well as periodontitis (29). There are several treatment strategies in order to eliminate or control PD. These include mechanical plaque control, the use of chemicals and herbal drugs (30). The mechanical method is unpleasant and painful and the chemical method, which is based on the systemic antibiotics therapy, is associated with the risk of bacterial resistance. However, natural plant compounds' ability to modulate immune inflammatory response has evoked interest as alternates for many diseases, including periodontitis. 

Therefore, natural agents having antimicrobial and anti-inflammatory activities might be able to control the inflammatory diseases such as periodontitis. The flavonoid anethole has been shown to suppress inflammation by inhibiting the cellular responses induced by TNF (27). Anethole has been reported to exert local anesthetic (31), sedative (32), oestrogenic, anti-genotoxic and anti-tumor activities (33) with no or little toxic side effect. As mentioned above, herbal medicine principally has been used as traditional treatments for many human diseases, including infectious diseases (*e.g*. periodontitis). The natural plant-derived medicines used in folk medicine have been proven to be efficient in treating infections (34). Very recently has been reported that resveratrol, a polyphenolic compound found in several plants, may exert immunomodulatory effects on the host response and decreases periodontal breakdown and modulates local level of cytokines in an animal model of periodontitis (35). It has also been demonstrated that curcumin, *a component* of tumeric which is used as a dietary spice, exerts a potent anti-inflammatory activity against LPS-induced periodontal disease (36). 

In the present the anti-inflammatory effect of trans-anethole was evaluated on a rat model of LPS-induced periodontitis. Findings indicated that anethole particularly at 50 mg/Kg suppressed significantly both IL-1β and TNF-α when compared to LPS-treated rats. The suppressive effects of trans-anethole on LPS-induced pro-inflammatory cytokines (IL-1β and TNF-α) were almost similar as seen with ketoprofen, a nonsteroidal anti-inflammatory drug. It has already been reported that anethole is a potent inhibitor of TNF-α (Chainy et al., 2000). In agreement with our findings, Ponte and colleagues (2012) documented the inhibitory activity of anethole on paw edema induced by TNF-α (12). 

Although in the present work the mechanism responsible for the suppressive action of trans- anethole on IL-1β or TNF-α production was not assessed, it has been previously reported that trans- anethole modifies possibly the voltage-gated L-type Ca^2+^ channel and KCa^2+^ channels function (22, 37). The blockade of L-type Ca^2+^ channels has been shown to inhibit the LPS-induced inflammatory molecules production (38). L-type Ca^2+^ channels are members of voltage gated membrane ion channels which mediate Ca^2+ ^entry in many excitable and non-excitable cells, including immune cells (39). On the other hand, it has been experimentally demonstrated that K_Ca2+_ channel opener and Kv1.3 channel blockers exert anti-inflammatory effects (40). 

Besides the effects of anethole in suppressing the inflammatory responses possibly through modulation of ion channel functions, it has been shown that different participation of substance P, bradykinin, histamine and serotonin might be also involved in the anti-inflammatory effect of anethole (27). However, further studies are needed to elucidate the exact mechanism through which anethole exerts its anti-inflammatory effects in periodontitis.

In conclusion, the results of the present investigation suggest that anethole may have a potent inhibitory effect on periodontitis through suppression of pro-inflammatory molecules (*i.e*. IL-1β and TNF-α); therefore it could be a novel therapeutic strategy for periodontitis. However, further studies are needed using morphometric analysis to define the effect of anethole on bone loss and molecular techniques to investigate the exact mechanism through which anethole exerts its anti-inflammatory actions. 
